# Increased Risk of High Body Fat and Altered Lipid Metabolism Associated to Suboptimal Consumption of Vitamin A Is Modulated by Genetic Variants rs5888 (*SCARB1*), rs1800629 (*UCP1*) and rs659366 (*UCP2*)

**DOI:** 10.3390/nu12092588

**Published:** 2020-08-26

**Authors:** Sebastià Galmés, Andreu Palou, Francisca Serra

**Affiliations:** 1 Laboratory of Molecular Biology, Nutrition and Biotechnology, NUO Group, Universitat de les Illes Balears, 07122 Palma, Spain; s.galmes@uib.cat (S.G.); francisca.serra@uib.es (F.S.); 2 CIBER de Fisiopatología de la Obesidad y Nutrición (CIBEROBN), 28029 Madrid, Spain; 3 Institut d’Investigació Sanitària Illes Balears (IdISBa), 07120 Palma, Spain; 4 Alimentómica S.L., Spin-off n.1 of the University of the Balearic Islands, 07121 Palma, Spain

**Keywords:** personalized nutrition, dietary vitamin A, obesity, body fat, UCP, retinoic acid, PBMC

## Abstract

Obesity is characterized by an excessive body fat percentage (BF%). Animal and cell studies have shown benefits of vitamin A (VA) on BF% and lipid metabolism, but it is still controversial in humans. Furthermore, although some genetic variants may explain heterogeneity in VA plasma levels, their role in VA metabolic response is still scarcely characterized. This study was designed as a combination of an observational study involving 158 male subjects followed by a study with a well-balanced genotype–phenotype protocol, including in the design an ex vivo intervention study performed on isolated peripheral blood mononuclear cells (PBMCs) of the 41 former males. This is a strategy to accurately identify the delivery of Precision Nutrition recommendations to targeted subjects. The study assesses the influence of rs5888 (*SCARB1*), rs659366 (*UCP2*), and rs1800629 (*UCP1*) variants on higher BF% associated with suboptimal VA consumption and underlines the cellular mechanisms involved by analyzing basal and retinoic acid (RA) response on PBMC gene expression. Data show that male carriers with the major allele combinations and following suboptimal-VA diet show higher BF% (adjusted ANOVA test *p*-value = 0.006). Genotype–BF% interaction is observed on oxidative/inflammatory gene expression and also influences lipid related gene expression in response to RA. Data indicate that under suboptimal consumption of VA, carriers of VA responsive variants and with high-BF% show a gene expression profile consistent with an impaired basal metabolic state. The results show the relevance of consuming VA within the required amounts, its impact on metabolism and energy balance, and consequently, on men’s adiposity with a clear influence of genetic variants *SCARB1*, *UCP2* and *UCP1*.

## 1. Introduction

Obesity is a metabolic disease characterized by excessive fat accumulation which can promote the development of associated disorders, such as diabetes or cardiovascular events, resulting in an increased risk of mortality and considerable public health costs [1]. Furthermore, its prevalence has not stopped growing over the last few decades [2]. Meanwhile, poor consumption of vitamin A (VA) has been widely related to vision problems [3] and immune system alterations [4], but also with a higher prevalence of obesity [5,6] and fat accumulation [7,8].

Therefore, scientific evidence suggests a link between dietary VA and the regulation of energy balance. Dietary intake of vegetables and fruits facilitates the availability of pro-VA in the form of β-carotene (BC), which is enzymatically converted to retinaldehyde and then irreversibly oxidized to retinoic acid (RA) [9]; whereas animal sources provide retinol, mainly as retinyl esters with fatty acids, which is metabolized in the cells to retinaldehyde at a higher efficiency than BC [10]. The main active form of VA is RA which has the capacity to influence the expression of key genes related to lipid and energy homeostasis in mammals [11,12], actively participating in the modulation of adipocyte differentiation, lipogenesis/lipolysis, thermogenesis, and fat oxidation [11,12,13]. Furthermore, active VA metabolites increase fatty acid oxidative metabolism [14]. The main core of studies on supplementation with BC have been based on the assessment of BC antioxidant action [15], while studies aimed at inducing weight loss or body fat reduction using carotenoid supplementation are still scarce. Nevertheless, in a recent small double-blind randomized study performed on 17 children supplemented for 6 months with a combination of carotenoids, a reduction in parameters associated with adiposity and obesity was observed [16], in accordance with a recent meta-analysis linking carotenoids and VA with the occurrence of metabolic syndrome [17]. Although higher serum levels of carotenoids are inversely associated with metabolic syndrome, large interindividual variation as regards the metabolic conversion of dietary BC is also found [17].

In particular, genetic variants may support the foundations of interindividual variability in nutritional status and handling of VA. In fact, bioavailability of BC has been shown to be dependent on genes involved in postprandial chylomicron metabolism (ATP-binding cassette, subfamily A (*ABC1*, *ABCA1*); APOB; Transcription factor 7-like 2 (*TCF7L2*); and hepatic lipase (LIPC)) as well as in the uptake, absorption, and subsequent tissue management of BC (such as scavenger receptor class B, member 1 (*SCARB1*) and ATP-binding cassette, subfamily G, (*ABCG5*)) [18]. In addition, family studies have estimated that 30% of the variation in serum retinol is heritable [19], and a single nucleotide polymorphism (SNP) has already been related to plasma retinol levels [20]. Therefore, a core of evidence suggests the existence of genetic variants that may influence the metabolism of VA, either precursors or derived metabolites; although the underlying mechanisms are not totally understood. Furthermore, to date, no studies have assessed the metabolic impact of gene variants that may be predisposed to a greater risk of obesity in the case of following suboptimal intake of VA. In this research, three genetic variants that may determine the modulation of VA efficiency and metabolism were analyzed: rs5888 located on Scavenger Receptor Class B type 1 (*SCARB1*) which encodes the protein SR-B1, a multifunctional scavenger receptor involved in dietary/blood carotenoid cell uptake and transport [21]; rs1800592 associated with the Uncoupling Protein-1 (*UCP1*) with a key role in thermogenesis and fatty acid oxidation and inducible by carotenoids and retinoids [8,22]; and rs659366 on Uncoupling Protein-2 (*UCP2*) which is involved in oxidative cell status and is induced by vitamin A [8,23]. In addition, previous studies suggest that the presence of certain alleles of these SNPs could be related to differential bioactive compound transport or responsive capacity [24,25,26] (see details in Table S1).

Therefore, we conducted a study to obtain further insight on the role of these specific gene variants in the susceptibility to obesity under suboptimal VA status, which was performed in two sequential parts. First, the study analyzed the role of the three genetic variants in a Spanish population (Mallorca) in order to assess whether genetics could contribute to explain the greater rate of obesity associated with a suboptimal consumption of VA described in animal models. Then, ex vivo exposure of Peripheral Blood Mononuclear Cell (PBMC) samples to RA was performed to gain further insight into the mechanisms involved by analyzing the expression of a set of lipid and oxidative metabolism key genes.

## 2. Materials and Methods

### 2.1. Subjects

**Figure 1:**
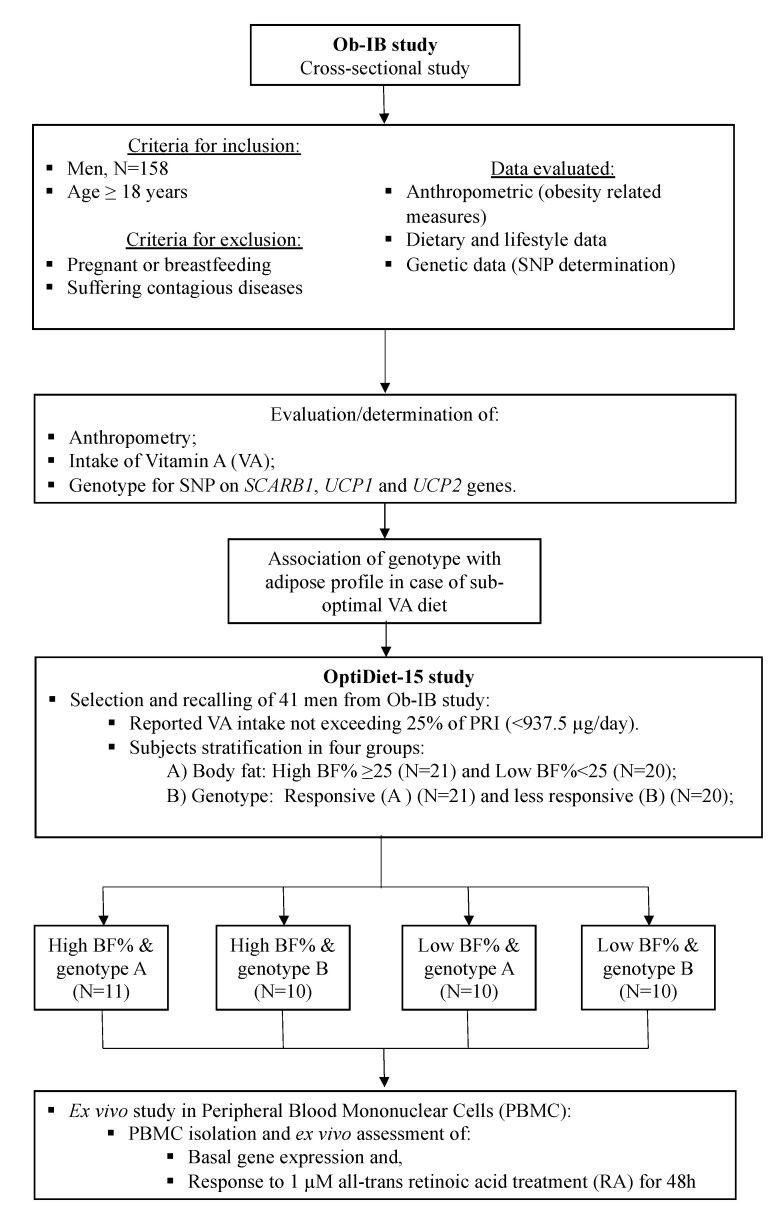
Flowchart diagram of the study. Flowchart diagram of the main steps of the study, including data sets recorded, criteria established for subject selection, number of subjects taken into account, and main procedures followed at each stage. PRI-Population Reference Intake, SNP-single nucleotide polymorphism.

Protocol was in accordance with the Declaration of Helsinki principles and approved by the ethics committee (Comitè d’Ètica de la Investig. de les Illes Balears, CEI-IB). Procedures and a flowchart are summarized in Figure 1. At the first step, information concerning specifications of the subjects, anthropometrics, and habitual intake, was collected from each participant, together with a saliva sample for genotyping (following the protocol defined at Ob-IB study (approval IB2009/13)). At the next step, we focused the study on the male cohort in order to characterize the relationship observed among adiposity and intake of retinol source foods. Subjects were selected following a “case–control” protocol and the design included an ex vivo intervention study (OptiDiet-15 study, IB2569/15) performed on isolated PBMCs of the former subjects—a selected subset of 41 men, taking into account their dietary, genetic, and anthropometric profile.

Specifically, the ninety men who did not meet nutritional requirements for vitamin A (<750 µg/day) were initially preselected for the OptiDiet-15 study and were stratified by genotype and body fat (BF). Around 60% of them were contacted again to ask for their wiliness to participate in the OptiDiet-15 study to characterize the effects of a low-VA diet associated with the presence of allele combinations of selected SNPs—rs5888, rs659366, and rs1800592. To minimize any bias, care was taken to select balanced groups concerning VA more responsive and less responsive genotypes (hereafter called Genotype A, *n* = 21; Genotype B, *n* = 20, respectively) and BF% (high BF% ≥ 25; *n* = 21; low BF% < 25; *n* = 20). Those subjects who were willing to participate were checked for diet and anthropometry to verify that the subjects still fulfilled the selection criteria, to update the information, and to further confirm their grouping. Incorporation into the different groups was managed in parallel to avoid imbalances. Eighty percent of the subjects confirmed suboptimal habitual intake of VA, and those not exceeding 25% of Population Reference Intake (PRI) (<937.5 µg/day) were considered suitable to be further analyzed. Recruitment finished when balanced groups with the desired genotype combinations and BF% were obtained.

A blood sample was taken for plasma determinations and PBMC extraction for ex vivo incubation and gene expression analysis.

### 2.2. Anthropometric Measures

Body fat percentage (BF%) was measured with a bio-impedance apparatus (OMRON BF306, Kyoto, Japan). The waist–hip (WHR) and the waist-to-height ratios (WtHR) were obtained by waist circumference/hip circumference and waist circumference/height, respectively. Body adiposity index (BAI) was obtained using the formula [27]: BAI = ((hip circumference/height^1.5^) − 18). Bicipital, tricipital, subscapular, supraspinatus, and abdominal skinfolds were measured using a Harpenden caliper (Baty International, Burgess Hill, UK).

### 2.3. Estimation of Dietary Intake

Dietary intake was assessed with a 24 h recall report (24RR) during individual face-to-face interviews. We collected up to three 24RR for each participant to ensure the quality of the data recorded, wishing to diminish the influence of random measurement error and to correct for day-to-day variation within subjects. The subject mean of this set of 24RRs was used as a proxy of habitual intake of VA. In the OptiDiet-15 cohort, dietary intake was characterized with two and three 24RRs, in 71% and 27% of the subjects, respectively. Concerning the 41 subjects finally characterized at the PBMC level, we reinforced the number of subjects with three 24RRs (31%) and analyzed 66% with two 24RRs.

In addition, we applied a standardized protocol aiming to minimize forgotten food items and correctly estimate portion sizes of foods. Therefore, intake data were collected during face-to face interviews. The researcher (S. Galmés) handled an image book containing habitual dishes and recipes which was used to help the individuals to form an accurate report of the last 24 h intake. In addition, the book also included different sizes either of single foods (as an apple or a slice of bread) or more complex dishes (spaghetti, paella, etc.). This was used to better define the amount of food eaten.

Then, this food information was transferred to the software DIAL that includes the composition of most Spanish foods and dishes and also allows the introduction of new food items and composition. The intake of each dietary ingredient was converted to energy and nutrient composition using the dietary software DIAL v2.0 (Alce-Ingeniería, Madrid, Spain) [28]. Total VA intake was determined (µg of retinol + (carotenoids with vitamin activity/6)). Population Reference Intake (PRI) by European Food Safety Authority (EFSA) for men was used as a cut-off point (750 μg/day) [29] to classify VA intake.

Individuals who reported taking supplements that may affect the nutritional status of vitamin A were not included in the analysis.

### 2.4. SNP Selection and Population Grouping According to Genotype

Genetic variants rs5888, rs659366, and rs1800592 located on *SCARB1*, Uncoupling Proteins 2 and 1 (*UCP2*, *UCP1*) genes, respectively, were selected because of their location on genes involved in VA absorption, handling, and/or metabolism and based on their response to bioactive compounds [13,22,30,31,32,33,34,35,36,37] (see Table S1 for a detailed summary).

In brief, the T variant of SNP on *SCARB1* is associated with decreased levels of its protein in vitro [38] and with lower levels of fat-soluble antioxidants in plasma [24]. Thus, it was hypothesized that the effect of the T allele (TT + TC genotypes) could be counteracted with high VA intake. Regarding rs659366 and rs1800592, T allele carriers and AA genotype, respectively, showed a greater anti-obesogenic response to bio-active compounds together with a higher expression of their respective genes [39,40,41]. Accordingly, a dominant model for *SCARB1* rs5888 and *UCP2* rs659366 (TT + TC vs. CC, for both of them) and a recessive model for *UCP1* rs1800592 (AA vs. AG + GG) were produced. Therefore, driving towards the hypothesis that subjects with T (rs5888), T (rs659366), and/or without G (rs1800592) would have a greater predisposition to respond more effectively to bio-active compounds, such as VA, and/or show major metabolic risk in the case of suboptimal intake.

According to this, the genotype grouping of subjects in both Ob-IB and OptiDiet-15 studies was performed according the presence of two or more”responsive” genotypes (TT or TC for rs5888; AA for rs1800592; and/or TT or TC for rs659366), tested as Genotype”Responsive” (A), in contrast with subjects having at most one of the responsive genotypes aforementioned, which were defined as Genotype B (less responsive).

### 2.5. DNA Extraction and Genotype Determination

Saliva samples were obtained following a standardized procedure. In brief, thirty minutes before sample collection, the subjects were requested to avoid eating, drinking, smoking, and chewing gum. Then, they were asked to spit into a collection tube until the liquid saliva reached 2 mL, without taking bubbles into account. Then, fresh saliva was immediately used to isolate genomic DNA or stored in adequate conditions (4 °C) until DNA extraction was performed. Isolation of genomic DNA was carried out using the commercial kit High Pure PCR template Preparation Kit (Roche, Basel, Switzerland). Genotyping was performed by qPCR (LightCycler^®^480 FastStart DNA, Roche, Basel, Switzerland), FastStart master mix (Roche, Basel, Switzerland), and specific-SNP (Tib Molbiol, Berlin, Germany) following the conditions described elsewhere [42].

### 2.6. Blood Sample Collection, PBMC Isolation, and Ex Vivo Treatment

Peripheral Blood Mononuclear Cells (PBMCs) constitute a source of biomarkers of metabolic status as well as a potential ex vivo system to test food bioactives’ efficacy—easier to obtain than samples of other tissues, such as adipose tissue or liver [43]. Therefore, venous blood (20–25 mL) was collected from volunteers early in the morning in the fasting state using vacutainer-EDTA tubes. PBMCs were isolated by density gradient using Ficoll-Paque Plus (Healthcare Bio Science, Barcelona, Spain) following the protocol described in Cifre et al., 2016 [44]. Finally, 1 × 10^6^ cells were activated with 0.5 × 10^6^ of CD3/CD28 magnetic beads (Life Technologies, Madrid, Spain) and maintained in suspension in a RPMI-1640 medium, with fetal bovine serum (10%), L-glutamine (1%), penicillin (100 units/mL) streptomycin (100 µg/mL), and DMSO (control cells) or all-trans retinoic acid (RA) (1 µM) for 48 h at 37 °C and 5% CO_2_. Medium components and treatments were purchased from Sigma-Aldrich (St Louis, MO, USA).

### 2.7. RNA Extraction and Gene Expression Analysis

After the incubation period, total RNA was isolated using Direct-zol RNA Mini-Prep (Zymo Research Corp, Irvine, CA, USA). mRNA concentration was determined by a spectrophotometric-based system (NanoDrop 1000, ThermoFisher Scientific, Waltham, MA, USA). RNA purity was assessed by the absorbance ratios at 260/280 and 260/230 nm. Randomized quality controls were introduced to check RNA integrity. This was assessed performing a 1% agarose gel electrophoresis. RNA (0.05 µg) were transcribed into cDNA using a highly sensitive first-strand cDNA synthesis kit (iScript cDNA, Bio-Rad Laboratories, Madrid, Spain). Real-time PCR was performed for each RT product to determine mRNA expression using the Power SYBR Green PCR Master Mix (Applied Biosystems, Madrid, Spain) as described in a previous publication [44]. All primers were purchased from Sigma Genosys (Sigma-Aldrich Química SA, Madrid, Spain). Two common housekeeping genes on PBMCs were analyzed—Elongation factor 1-alpha 1 (EF1a1) and Ribosomal Protein Large P0 (*RPLP0*).

The threshold cycles (Cts) were obtained using the StepOne v2.0 software (Applied Biosystems, Madrid, Spain). Then, the Cts of each analyzed gene were normalized against the housekeeping gene RPLP0 from the same sample. This housekeeping gene gave threshold cycles closer to the ones of the genes of interest. Then, relative PBMC gene expression was calculated as a percentage referring to the gene expression of control cells from genotype A and low BF% subjects (considered as 100%). Gene expression was determined for a set of genes of relevance in lipid metabolism and/or with a role in the mediation of cellular oxidative stress. Therefore, mRNA levels of *LXRA*, *SOD2*, *SLC27A2*, *SREBP1C*, *SCARB1*, *CEBPB*, *RXRA*, *CPT1A*, and *UCP2* were analyzed.

### 2.8. Statistical Analyses

Descriptive data are generally presented as the mean and standard deviation. Data parametricity was assessed by the Kolmogorov–Smirnov and Levene tests, otherwise variables were log_10_ transformed. ANOVA tests adjusted for confounding variables (detailed in footnotes of each table or figure) were used to compare the means between groups. Appropriate intake of macro/micronutrients was assessed by a *t* test for a single sample by comparison with the recommendations set for adult men [29,45]. Linear regression analyses adjusted for age using the level of VA intake as a dichotomous (LI and RI) independent variable and body adiposity measures (BF% and BAI) were performed to confirm the nutrigenetic relationship between dietary VA and adiposity associated with the genotype. Two-way ANOVA tests were performed to evaluate the genotype–BF% interaction effects on PBMC basal gene expression followed by a least significant difference (LSD) posthoc comparison. ANOVA tests were adjusted for the main covariates (or combinations of covariates) that could be causing biases between the specific groups that were compared. In this regard, the main confounding variable for genotype groups was total energy intake, and these comparisons were adjusted for this covariant; the main confounding variable detected relative to the BF% groups was age. Consequently, the ANOVA tests to compare these groups were adjusted for age. Therefore, interaction ANOVA tests comprising genotype–BF% groups were adjusted for both confounding variables. The RA treatment effect was evaluated by a Student’s *t* test for paired data by comparing control cells with RA-treated ones. The genotype–BF% interaction on RA treatment effects was assessed by ANOVA for paired data. All statistical analyses were performed using the SPSS v25.0 (IBM, Chicago, IL, USA), and the threshold of significance was defined at *p* < 0.05 for all analyses.

## 3. Results

### 3.1. Evaluation of VA Intake Level and Genotype Impact on Adiposity in Men of the Ob-IB Study

The main characteristics of the male cohort in the Ob-IB study are summarized in Table 1. Volunteers showed an anthropometric profile of overweight: body mass index (BMI) = 26.5 ± 5.0 kg/m^2^ and waist–hip (WHR) = 0.93 ± 0.07. Dietary energy intake was quite similar (2231 kcal/day) to that reported in the ANIBES study for adult men (1966 kcal/day) [46] and close to actual recommendations for moderately sedentary adult men [47]. Analysis of the dietary profile showed an imbalanced diet except for protein consumption, which was within the EFSA recommendations for the general population (15.9%; *p* = 0.075 vs. recommended 15–20%). Thus, fat energy contribution was higher than recommended (37.2%; *p* = 0.005 vs. recommended 20–35%) at the expense of carbohydrates (42.6%; *p* = 0.003 vs. recommended 45–60%). Accordingly, intake of fiber was also below recommendations (22.8 g/day; *p* < 0.001 vs. recommended > 25 g/day), but simple sugars exceeded the advisable limit of 10% (16.2%; *p* < 0.001). Concerning the quality of dietary fat, only monounsaturated fatty acids (MUFA) were within recommendations (15.4%; *p* = 0.439 vs. recommended 15–20%), whereas saturated fatty acids (SFA) were higher (12.4%; *p* < 0.001 vs. recommended < 10%), and polyunsaturated fatty acids (PUFA) were below the recommended intake (6.1%; *p* < 0.001 vs. recommended 7–10%). The dietary profile found, including total calories and energy contribution of macronutrients, was very similar to that observed in the Spanish male population [46].

Allele frequencies of the SNPs studied were in general accordance with the frequency of the European population (available at 1000Genomes (http://www.internationalgenome.org)). Genotypes including the allele identified as potentially responsive to VA showed the highest frequency in the Ob-IB population: 66.5% of TT/TC for rs5888; 51.3% AA for rs1800592; 65.2% TT/TC for rs659366 (Table 1).

Concerning intake of VA, the reported daily consumption showed an average intake that was 48% higher than the Population Reference Intake for adult men [29] (PRI: 750 µg/day; *p* = 0.058) although with a high range of variability (range: 110–45,239 μg/day) (Table 1). Forty three percent of volunteers (irrespective of the genotype) reported intakes comprising PRI or higher (≥750 µg/day) and were classified as the recommended intake (RI) group, whereas the rest, with VA intake < 750 µg/day, were classified as the low intake (LI) group. Subsequent stratification of subjects according to the genotype (responsive, A versus less responsive, B) allowed a link between genetic background and dietary VA on adiposity to be shown. BF% of Genotype B was 25.8% and 23.8% in the LI and RI groups, respectively. Whereas in Genotype A, BF% was 25.9% in the LI and 22.1% in the RI groups (*p* = 0.006) (Table 2), suggesting that the allele load in Genotype A could constitute a good proxy of genetic variants driving physiological response to VA, particularly in the context of energy balance and its interaction with adiposity. Linear regression analyses using the level of VA intake (LI and RI) and BF% after adjusting by age, confirmed the nutrigenetic relationship between dietary VA and propensity to obesity associated with the genotype. Genotype A individuals were significantly associated with lower BF% (β = −4.11, *p* = 0.006) and BAI (β = −1.99, *p* = 0.029) when fulfilling dietary VA recommendations (RI group) taking low VA intake as the reference group, whereas this was not the case for Genotype B subjects (BF% (β = −0.10, *p* = 0.972) and BAI (β = −0.28, *p* = 0.988)) in comparison with the low VA intake group (Table 3). In agreement with the hypothesis, data pointed out that Genotype A would be specifically susceptible to improving body composition by increasing VA consumption to current recommendations.

### 3.2. Assessment of the Influence of Suboptimal VA Intake, Genotype, and Adiposity on PBMC Metabolism

Aiming to further characterize the metabolic relevance of genotype on BF%, particularly under the influence of suboptimal VA intake, the OptiDiet-15 study was undertaken in the following steps. Subjects who had initially reported a VA intake below recommendations (<750 µg/day) were contacted again and rechecked. Eighty percent of them confirmed suboptimal habitual intake of VA, and subjects not exceeding 25% of PRI (<937.5 µg/day) were considered suitable to be further analyzed. Therefore, a case–control design, including balanced groups in terms of genotype (A or B) and BF% (cut off at 25%), was accomplished (*n* = 41) to study gene expression in isolated PBMC as well as to analyze PBMC ex vivo response to RA.

Anthropometric and dietary characteristics are shown in Table 4. No major anthropometric differences were found between genotypes within the same group of body fat. However, concerning dietary characteristics in High-BF% groups, Genotype A subjects reported higher energy intake than those belonging to Genotype B (*p* = 0.007), involving greater energy from MUFA (19.1%; *p* = 0.003) and higher protein intake (94.1 g/day; *p* = 0.014) in comparison to subjects with Genotype B (13.7% of MUFA; 72.1 g protein/day). In contrast, in Low-BF% groups, Genotype A subjects showed lower PUFA intake (4.9%) than Genotype B subjects (7.0%, *p* = 0.027).

**Figure 2:**
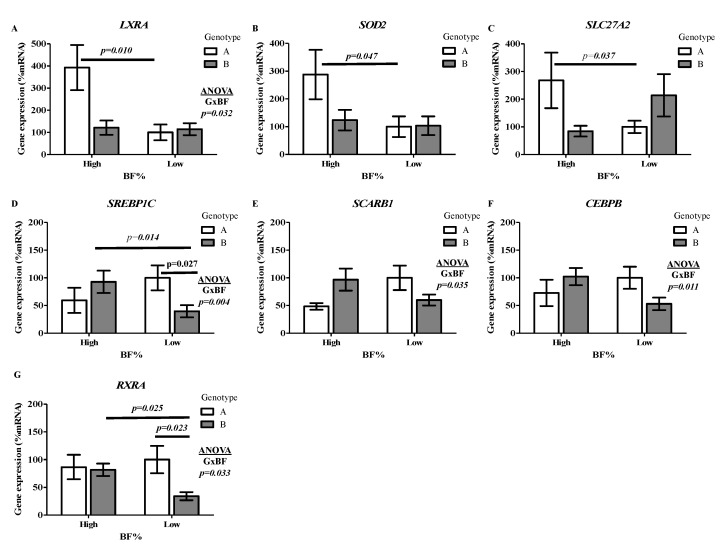
Gene expression under basal conditions in Peripheral Blood Mononuclear Cells. Gene expression under basal conditions in Peripheral Blood Mononuclear Cells (PBMCs) of subjects according to genotype (A/B) and BF% level (High/Low) groups. Data correspond to mRNA levels of genes encoding transcription factors involved in cellular response to retinoic acid, lipid, and energy metabolic homeostasis: (**A**) Liver X Receptor alpha (LXRA), (**B**) Superoxide dismutase 2 (SOD2), (**C**) Very long-chain acyl-CoA synthetase (SLC27A2), (**D**) Sterol Response Element Binding Protein 1c (SREBP1C), (**E**) Scavenger Receptor Class B Member 1 (*SCARB1*), (**F**) CCAAT/enhancer-binding protein beta (CEBPB), and (**G**) Retinol-X Receptor α (RXRA). The mRNA levels were normalized to RPLP0 and expressed in relation to the expression found in Genotype A and Low BF% which was set at 100%. The statistical analysis of genotype–BF% interaction was carried out by ANOVA adjusted for total energy intake and age; GxBF indicates *p* < 0.05. Differences between genotype groups were established by an ANOVA test adjusted for total energy intake. Differences between BF% groups were analyzed by an ANOVA test adjusted for age.

To assess the impact of genotype and BF% on cell metabolism, the expression of genes involved in lipid homeostasis was analyzed in PBMCs under basal conditions. In High-BF% subjects, the influence of Genotype A was reflected by the increased expression of Liver X receptor alpha (*LXRA*) (*p* = 0.010) (Figure 2A), Superoxide Dismutase-2 (*SOD2*) (*p* = 0.047) (Figure 2B), and Acyl-CoA synthase (*SLC27A2*) (*p* = 0.037) (Figure 2C), in comparison with Low-BF% subjects with the same genotype. In contrast, High-BF% subjects with Genotype B showed increased mRNA levels of Sterol Response Element Binding Protein 1c (*SREBP1C*) (*p* = 0.014) (Figure 2D) and Retinoid X Receptor alpha (*RXRA*) (*p* = 0.025) (Figure 2G) in comparison with Low-BF% and the same genotype. Furthermore, the specific influence of genotype was observed on *SREBP1C* (*p* = 0.027) and *RXRA* (*p* = 0.023) expression only in Low-BF%, in which Genotype A presented a higher expression than Genotype B. Furthermore, gene expression showed interaction effects between genotype and adiposity. Specifically, interactions were shown on gene expression of *LXRA* (*p* = 0.032) (Figure 2A), *SREBP1C* (*p* = 0.004), Scavenger Receptor class B member 1 (*SCARB1*) (*p* = 0.035), CCAAT/enhancer-binding protein beta (*CEBPB*) (*p* = 0.011), and *RXRA* (*p* = 0.033) (Figure 2D–G).

**Figure 3:**
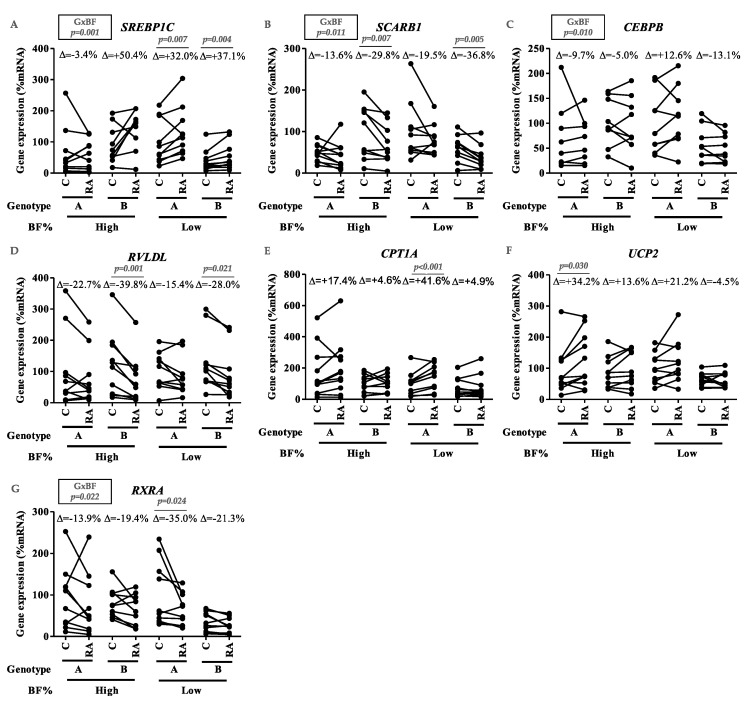
Effect of all-trans retinoic acid treatment on mRNA expression in Peripheral Blood Mononuclear Cells of Genotype (A/B) and BF% level (High/Low). Effect of all-trans retinoic acid (RA, 1 µM) treatment on mRNA expression in Peripheral Blood Mononuclear Cells (PBMCs) of subjects according to genotype (A/B) and BF% level (High/Low) groups. Data correspond to mRNA levels of (**A**) Sterol Response Element Binding Protein 1c (SREBP1C), (**B**) Scavenger Receptor Class B Member 1 (SCARB1), (**C**) CCAAT/enhancer-binding protein beta (CEBPB), (**D**)Very Low Density Lipoprotein Receptor (RVLDL), (**E**) Carnitine palmitoyltransferase I alpha (CPT1A), (**F**) Uncoupling Protein 2 (UCP2), and (**G**) Retinol-X Receptor α (RXRA). The % increase (Δ) was obtained by the difference between the mean of the gene expression in RA-treated cells and the mean of gene expression under baseline conditions (C) in each experimental group. Statistical assessment of the effect of treatment on each experimental group was estimated using a paired t-test. Genotype–BF% interaction regarding the effect of treatment was assessed using repeated measures ANOVA (GxBF, *p* < 0.05).

To obtain further insight into the molecular mechanisms involved, cellular response was analyzed in PBMCs incubated with RA. Interestingly, the interactive effects of genotype–BF% were found on gene expression of *SREBP1C* (*p* = 0.001), *SCARB1* (*p* = 0.011), *CEBPB* (*p* = 0.010) (Figure 3A–C), and *RXRA* (*p* = 0.022) (Figure 3G). In addition, RA treatment caused specific increases in *CPT1A* (*p* < 0.001) and *UCP2* (*p* = 0.030) (Figure 3E,F) in Genotype A subjects, with Low-BF% and High-BF%, respectively. Meanwhile, in Genotype B individuals, *SCARB1* (High-BF%, *p* = 0.007; Low-BF%, *p* = 0.005) and *RVLDL* (High-BF%, *p* = 0.001; Low-BF%, *p* = 0.021) expression was decreased by RA regardless of BF% (Figure 3B,D).

Concerning the gene expression of transcription factors, RA incubation decreased mRNA levels of *RXRA* (*p* = 0.024) (Figure 3G) in Genotype A and Low-BF% subjects and increased *SREBP1C* gene expression in Low-BF% (Figure 3A), regardless of the genotype (Genotype A, *p* = 0.007; Genotype B, *p* = 0.004). Altogether, data on gene expression showed the functional impact of the allelic load considered in handling VA and disclosed the cellular adaptations to RA delivery depending on BF%.

## 4. Discussion

Although a recent study underlined that different carotenoids in serum and adipose tissue in humans are associated with metabolic benefits, such as improvement in insulin sensitivity in both liver and adipose tissue [8,48], direct evidence in humans concerning the effects of dietary VA or carotenoid supplementation on body adiposity is limited [15]. Furthermore, it has been shown that variants in genes involved in carotenoid absorption could be responsible for high interindividual variability in their bioavailability [18,25,49].

Thereby, our findings show that three genetic variants, which have not been previously related to fat accumulation, might be predisposed to greater adiposity depending on the amount of VA intake; although we cannot rule out other genes or genetic variants also being relevant, particularly depending on population characteristics (e.g., sex, age, or lifestyle). However, the role of SNPs and the functional assessment carried out may contribute to providing further insight into the mechanisms involved in this approach. Specifically, allele combinations performing the responsive Genotype (A), involving T allele for rs5888 (*SCARB1*) and rs659366 (*UCP2*), respectively, and/or the absence of G for rs1800592 (*UCP1*), were associated with a higher BF% in individuals reporting suboptimal VA intake. In contrast, optimal consumption of VA was associated with normal BF%, suggesting that Genotype A may contribute to a more efficient adipose metabolism in a way that depends on dietary VA.

The rs5888 is located on exon 8 of the *SCARB1* gene and entails a synonym amino acid exchange (A350A) in SR-B1 protein, causing splicing activity alterations [50]. Interestingly, T allele related to a better adipose profile under optimal VA consumption in our study has been previously associated with a lower risk of coronary heart disease [51] and lower levels of plasma triglycerides, specifically in men [52]. Moreover, SR-B1 is a multifunctional scavenger involved in carotenoid uptake from diet [21], and the presence of genetic variants may change its effectiveness.

The rs659366 is located on the *UCP2* gene promoter and has a crucial role in ROS dissipation [37]. The T allele has been associated with higher *UCP2* expression and energy expenditure [41], lower risk of obesity [53], and lower Homeostatic Model Assessment (HOMA) index [54]. However, to our knowledge, this is the first report showing its functional relationship with VA intake.

The rs1800592 is located on the *UCP1* gene promotor, whose protein plays a role in mitochondrial thermogenesis by uncoupling oxidative phosphorylation and, therefore, diminishing the synthesis of ATP from nutrients [13]. The VA and UCP1 activity relationship is well documented in cell cultures [34] and rodents [22]: VA enhances fatty acid oxidation and promotes UCP1-associated thermogenesis. Major allele of rs1800592 (A) is associated with higher *UCP1* expression and greater thermogenic capacity [39,40] which confers differential outcomes to bio-active compounds with anti-obesogenic effects [25,55]. This would fit with our findings of the genotypes of subjects, who under optimal VA intake show leaner phenotype.

Therefore, aiming to characterize in further detail the metabolic consequences of adiposity associated with suboptimal VA intake and the role played by the genotype, a novel methodological approach was carried out following an ex vivo study in blood cells from selected individuals. Peripheral Blood Mononuclear Cells (PBMCs) are in a continuous interplay with the tissues, including adipose tissue depots, express key hormones involved in energy homeostasis and body weight control (leptin, visfatin, ghrelin), and respond to metabolic challenges. In addition, PBMCs are readily accessible, the sample collection is less invasive than adipose specimens, and similarities in gene expression between PBMCs and white adipose tissue have been shown under different nutritional status and metabolic challenges [43]. Despite the fact that previous results from our research group do not show differences between PBMC populations between normal weight and obese subjects [44], some studies indicate that the lymphocyte fraction may be altered in obesity [56,57]. Therefore, there is the possibility that the differences observed that the baseline expression of the analyzed genes between Low-BF% vs. High-BF% experimental groups could be influenced by differential lymphocyte proportions. For this reason, each genotype group includes its respective group with both Low and High-BF% representation. Furthermore, PBMC fraction is a tool increasingly used in nutrition and obesity research as a source of transcriptomic biomarkers, because its capacity to reflect the homeostatic state of key tissues and different blood cell count proportions in obesity is assumed to be one of the causes of transcriptomic alterations [58].

Furthermore, the possibility that obesity may change the proportion of immune cell types can be discarded as the proportion of lymphocytes and monocytes in PBMC samples do not differ between normal-weight and overweight/obese men. Therefore, we may assume that the baseline gene expression differences associated to fat content are not due to differences in the immune cell type proportion. Therefore, PBMCs have been widely used and are considered a very useful tool in testing the efficacy of bioactive compounds in humans [43,44] as well as in nutrigenomics and transcriptomic based studies [42,59]

The overexpression of the genes *LXRA*, *SLC27A2*, and *SOD2* specifically found in High-BF% individuals with Genotype A would be indicative of altered metabolic status, reflected in their PBMCs, and resulting from the interaction of the genetic baggage with the low intake of VA. The *LXRA* gene codes for a transcription factor that exerts an enhancing role of the reverse transport of cholesterol and mediates inflammatory process in macrophages [60]. *SLC27A2* encodes a key enzyme that plays an important role in both lipid biosynthesis and fatty acid degradation [61]. Furthermore, its overexpression in rat PBMCs has been proposed as an early marker of overweight development, particularly related to inadequate diets [62]. *SOD2* is a member of the superoxide dismutase family, involved in the dissipation of ROS, which would confer protection against the cellular damage caused by oxidative stress and pro-inflammatory cytokines [63], raising its expression in elevated oxidative stress environments [64] in an attempt to prevent cell death [63]. Subsequently, increased expression of *LXRA* and *SLC27A2* in Genotype A individuals with High-BF% could be an indicator of lipotoxicity [65,66]. Altogether, the data reflect a compromised metabolic status that is in accordance with the findings from the observational study, in which men with Genotype A and low vitamin A intake showed greater risk of being overfat.

Additionally, genotype–BF% interactive effects were found regarding the expression of three genes involved in fat metabolism and lipid homeostasis. *SREBP1C* is a key regulator of cholesterol and fatty acid metabolism [67], *SCARB1* is a gene involved in the cellular transport of cholesterol [30], and *CEBPB* is a transcription factor that coordinates regulatory networks for inflammation and lipid metabolism in macrophages and adipocytes [68]. Thus, their gene expression would be conditioned by the adiposity of the subjects, their genetic load, and would affect cell lipid homeostasis.

Aiming to characterize the metabolic flexibility, PBMCs of the subjects were incubated with retinoic acid (RA). PBMCs from Genotype A subjects (which showed lower adiposity in the case of optimal VA consumption) treated with RA presented greater increases in *CPT1A* and *UCP2* expression than in Genotype B subjects (whose VA intake level was not related to BF%), suggesting greater the induction of mitochondrial fatty acid oxidation [14,33] (Figure 3). The increased mitochondrial activity would not enhance the *NFKB* mediated inflammatory response [69], inasmuch as *NFKB* expression decreased in these subjects (data not shown).

Further, genotype seems to influence lipid metabolism and cell fat management, since RA was associated with a decreased expression of *SCARB1* and *RVLDL*—both genes involved in the uptake of fat-soluble compounds—which were only significant in Genotype B regardless of BF% (Figure 3). In addition, the expression of lipid key genes (*SREBP1C*, *SCARB1*, and *CEBPB*) reflected the interactive effect of genotype and BF%. Thus, the combined effect of these two factors (body fat and genotype) conditions lipid homeostasis, as well as exerts a modulating effect of RA on the expression of key genes in PBMCs.

The influence of BF% on the response to RA was also noted, since RA caused an increase in the expression of *SREBP1C* in cells from individuals with Low-BF% of both genotypes (Figure 3A) and a decreased *RXRA* expression in Genotype A with Low-BF% but not in Genotype B individuals (Figure 3G). The differential activation/inhibition of response elements and transcription factors related to lipid and VA metabolism could be key to explaining the differential effects of RA, depending on genotype and BF%, by regulating a large network of genes involved in cell activity and metabolism [60,70]. In this sense, high adiposity could short-circuit some pathways related to the bioactive function of RA. The attenuation of the beneficial effects of certain bioactives on health due to the presence of obesity has been previously reported in prior studies using PBMCs. In particular, overweight/obesity alters the anti-inflammatory response to polyunsaturated fatty acids [44]. In addition, our previous results also indicate a synergistic effect of excess body weight with the presence of specific genetic variants, modulating the inflammatory status [42].

In summary, subjects following suboptimal consumption of dietary VA with a responsive Genotype (A) were related to a higher risk of adiposity, in contrast with Genotype B subjects that did not show such a relationship between VA intake and body fat content. Furthermore, body adiposity degree, influenced by genotype characteristics, was a conditioning factor of gene expression under both baseline conditions and in response to RA. In PBMCs from suboptimal VA consumers, Genotype A showed increased *LXRA*, *SLC27A2*, and *SOD2* expression, especially in individuals with High-BF%, which was consistent with an impaired basal metabolic state. Interestingly, gene expression after incubation with RA was found to drive an improvement in the cellular status, particularly in Low-BF% (by increasing *CPT1A* and *UCP2*). In contrast, gene expression in Genotype B appeared less sensitive to BF% in basal conditions and also after incubation with RA. In unstimulated cells, gene expression was in accordance with a better metabolic state in comparison with Genotype A subjects. Hence, RA treatment led to decreasing the expression of key genes for the uptake/management of fat-soluble substances, such as *SCARB1* and *RVLDL*, in comparison with Genotype A.

However, there may be some limitations in this study that should be taken into account, such as the relative small sample size; the multiple testing performed on gene expression; and the potential effect of other environmental confounders, socio-economic factors, or genetic variants not considered.

## 5. Conclusions

Obesity is a multifactorial disorder; thus, its treatment and prevention are masked by several factors, including genetics and lifestyle factors [71]. In the present study, we show that suboptimal consumption of vitamin A is related to an increased risk of body fat accumulation in genetically predisposed individuals, who would represent approximately 38% of the individuals in the studied population. The results show the relevance of consuming VA within the required amounts, its impact on metabolism and energy balance, and consequently, on men’s adiposity with a modulating influence of genetic variants of *SCARB1*, *UCP2*, and *UCP1*.

Finally, the combination of an observational study together with a genotype–phenotype ex vivo intervention on PBMCs seems to be an effective strategy to contribute to Precision Nutrition. The study of genetics together with the characterization of gene–nutrient–phenotype interactions in a well-balanced study design facilitates personalized dietary and lifestyle advice and promotes healthier status through a focused and integrated approach.

## Figures and Tables

**Table 1:** General anthropometric dietary and genetic characteristics of male Ob-IB study participants ^1^.

Male Subjects (Ob-IB Study) (*n* = 158)	Mean	SD	
Age (years)	37	17	
Anthropometric measures			
Height (cm)	175	7.59	
Weight (kg)	80.9	15.5	
Hip (cm)	98.6	10.1	
Waist (cm)	92.0	14.8	
WHR	0.93	0.07	
BAI	24.8	5.03	
BMI (kg/m^2^)	26.5	5.04	
BF%	24.5	8.13	
Skinfolds (mm)			
Bicipital	7.27	4.52	
Tricipital	11.1	5.62	
Subscapular	14.1	6.59	
Supraspinatus	17.2	8.98	
Abdominal	21.7	9.84	
Dietary parameters			Recommendation
Energy intake (kcal/day)	2231	521	2000–2600
Carbohydrate (g/day)	237 (42.6%)	80.5	45–60% *
Fat (g/day)	92.1 (37.2%)	31.5	20–35% *
Proteins (g/day)	88.4 (15.9%)	26.1	15–20%
Fiber (g/day)	22.8	9.08	> 25 g/day *
Vitamin A (µg/day)	1113	3718	750 ^#^
Genetic features		Ob-IB (%)	1000genomes (%)
rs5888 (*SCARB1*)	TT + TC	66.5	70.6
	CC	33.5	29.4
rs659366 (*UCP2*)	TT + TC	65.2	61.0
	CC	34.8	39.0
rs1800592 (*UCP1*)	AA	51.3	58.1
	AG + GG	48.7	41.9

^1^ All values are the mean and SD (standard deviation). Dietary data are based on the analysis of a set of up to three dietary recalls of 24 h, and reported means are compared with current European Food Safety Authority (EFSA) recommendations. Statistical differences between Ob-IB study intakes and recommendations were assessed by a single sample t-test. * *p*-value < 0.05; ^#^ 0.05 < *p* value < 0.06. Genetic frequencies of the Ob-Ib study and 1000Genomes (for European populations) database are expressed as % for each genotype. Abbreviations: WHR (waist–hip ratio); BAI (body adiposity index); BMI (body mass index); BF% (body fat percentage).

**Table 2:** Anthropometric and dietary parameters by genotype group (A, Responsive to VA; B, Less responsive to VA) and Vitamin A (VA) intake level (LI, Low Intake < 750 µg/day; RI, recommended intake ≥ 750 µg/day) ^1^.

Variables	Genotype VA Responsive (A) (*n* = 106)								*p*-Value	Genotype Less VA Responsive (B) (*n* = 52)								*p*-Value	GxVA Interaction (*p*-Value)
	Low VA Intake (LI) (*n* = 60)				Recommended VA Intake (RI) (*n* = 46)					Low VA Intake (LI) (*n* = 30)				Recommended VA Intake (RI) (*n* = 22)					
	Mean	SD	Min	Max	Mean	SD	Min	Max		Mean	SD	Min	Max	Mean	SD	Min	Max		
Weight (kg)	81.8	17.3	49.5	140	79.3	14.1	47.9	122	0.352	81.2	15.3	60.0	133	81.1	14.1	61.9	127	0.611	0.383
WHR	0.93	0.08	0.70	1.12	0.92	0.08	0.72	1.11	0.430	0.95	0.08	0.81	1.12	0.91	0.05	0.85	1.01	0.466	0.905
BMI (kg/m^2^)	27.0	5.51	19.2	45.1	25.5	4.47	18.7	36.8	0.076	27.2	5.39	19.8	42.9	26.3	4.24	20.5	38.3	0.737	0.249
BF%	25.9	8.12	5.30	43.7	22.1	8.44	6.70	38.8	0.006	25.8	8.24	11.3	47.6	23.8	6.39	13.0	36.6	0.972	0.114
BAI	25.5	5.30	17.2	44.8	23.7	4.66	15.1	34.6	0.033	25.8	5.67	18.9	39.6	24.1	3.59	16.7	30.5	0.799	0.271
Bicipital SF	8.41	5.59	2.60	30.0	5.83	2.85	2.20	13.0	0.002	7.97	4.67	2.80	19.6	6.33	2.87	2.90	13.5	0.509	0.187
Energy (kcal/day)	2229	538	1253	374	2276	536	1384	4256	0.551	2058	456	1061	3132	2377	497	1615	3666	0.040	0.167
Carbohydrate (% EC)	43.7	10.5	21.8	73.7	41.1	9.64	20.5	57.6	0.194	43.2	9.18	30.0	64.3	42.1	10.2	19.9	63.3	0.508	0.739
Fat (% EC)	35.9	9.60	16.8	61.5	38.6	8.33	24.3	57.9	0.135	36.4	10.2	12.9	57.8	38.7	10.6	13.9	63.5	0.479	0.853
Proteins (g/day)	86.9	25.7	37.0	180	94.5	29.3	55.8	182	0.122	76.2	18.5	30.0	104	96.6	23.7	57.9	154	0.002	0.224
Fiber (g/day)	22.7	11.0	4.50	63.8	23.2	7.24	13.0	39.4	0.324	20.9	5.86	10.4	36.7	24.7	10.5	11.0	56.8	0.104	0.570
Vitamin A (µg/day)	458	148	110	744	2368	6762	751	45239	0.000	498	131	196	722	1118	342	764	1887	0.000	0.138
Retinol (µg/day)	238	115	2.10	527	1703	6829	22.3	45116	0.000	258	126	19.0	518	479	320	35.2	1625	0.020	0.558
ß-carotene (µg/day)	951	681	10.4	2999	3090	1930	15.6	9264	0.000	1140	788	120	3153	2795	1481	318	5166	0.000	0.682

^1^ All values are expressed as the mean, SD (standard deviation), min (minimum), and max (maximum). Genotype A (responsive): TT or TC for rs5888, AA for rs1800592, and/or TT or TC for rs659366; Genotype B (less responsive), having at most one of the responsive genotypes aforementioned. Abbreviations: VA (vitamin A); G (genotype); WHR (waist–hip ratio); BMI (body mass index); BF% (body fat percentage); BAI (Body Adiposity Index); SF (skinfold); and EC (energy contribution). Statistical assessment of the differences between LI and RI groups within each genotype was performed by an ANOVA test adjusted for age.

**Table 3:** Linear regression table of the effect of low vs. high vitamin A intake on adiposity depending on the Genotype A/B ^1^.

**Genotype A**	**Beta**	**SE**	***p*-Value**
BF%	−4.11	1.47	0.006
BAI	−1.99	0.92	0.033
**Genotype B**	**Beta**	**SE**	***p*-Value**
BF%	−0.10	1.75	0.972
BAI	−0.28	1.10	0.799

^1^ Linear regression analyses were performed using low VA intake as the reference group within each genotype group. Genotype A (responsive): TT or TC for rs5888, AA for rs1800592, and/or TT or TC for rs659366; Genotype B (less responsive), having at most one of the responsive genotypes aforementioned. Regressions were adjusted for age. Abbreviations: BF% (body fat percentage), BAI (Body Adiposity index), SE (Standard Error).

**Table 4:** Anthropometric and dietary parameters by body fat % (High BF ≥ 25% or Low BF < 25%) and genotype groups (A, Responsive to VA; B, Less responsive to VA) of the OptiDiet-15 cohort ^1^.

Variables	High Body Fat % (*n* = 21)								*p*-Value	Low Body Fat% (*n* = 20)								*p*-Value
	Genotype A (*n* = 11)				Genotype B (*n* = 10)					Genotype A (*n* = 10)				Genotype B (*n* = 10)				
	Mean	SD	Min	Max	Mean	SD	Min	Max		Mean	SD	Min	Max	Mean	SD	Min	Max	
Weight (kg)	93.4	23.0	63.6	152	82.0	10.8	68.8	106	0.179	68.7	9.92	52.7	85.0	74.0	8.14	63.3	86.7	0.176
WHR	0.93	0.10	0.78	1.11	0.85	0.09	0.66	1.01	0.077	0.87	0.09	0.78	1.11	0.83	0.06	0.73	0.90	0.249
BMI (kg/m^2^)	31.1	6.17	24.9	46.6	28.0	4.88	21.7	39.0	0.199	22.6	2.52	19.7	27.1	23.8	2.42	19.5	27.1	0.246
BF%	32.2	4.92	26.5	43.6	29.6	2.66	26.2	35.1	0.152	19.0	4.20	10.1	24.0	18.8	4.50	12.6	24.8	0.794
Bicipital SF	11.2	9.95	3.90	40.0	11.1	3.31	6.00	15.8	0.915	4.77	1.54	2.70	8.00	6.38	3.85	3.60	15.8	0.123
Energy (kcal/day)	2311	415	1880	3052	1860	212	1530	2130	0.007	2238	424	1253	2853	2047	276	1695	2500	0.226
Carbohydrate (% EC)	38.9	5.92	30.9	47.4	40.9	7.43	30.0	51.2	0.472	46.2	8.48	38.4	60.4	42.5	8.02	32.8	56.4	0.326
Fat (% EC)	40.7	4.72	33.8	47.8	35.6	9.81	12.9	46.4	0.190	33.9	7.41	20.9	46.0	39.0	8.33	25.1	48.3	0.208
Proteins (g/day)	94.1	21.4	54.1	122	72.1	11.6	44.5	85.4	0.014	80.2	20.9	53.6	112	82.0	10.3	66.1	94.30	0.922
Fiber (g/day)	19.9	5.78	14.7	35.5	19.4	5.53	8.10	25.7	0.599	21.6	7.51	12.0	32.0	18.6	5.75	10.1	25.50	0.511
Vitamin A (µg/day)	583	165	372	794	543	148	386	819	0.711	532	141	287	754	618	207	365	934	0.218
Retinol (µg/day)	231	140	59.9	429	254	121	28.3	397	0.227	250	86.4	132	436	280	106	111	497	0.553
ß-carotene (µg/day)	1612	1065	480	4300	1540	993	303	3450	0.791	1295	912	241	2999	1741	1160	308	4333	0.217

^1^ All values are expressed as the mean, SD (standard deviation), min (minimum), and max (maximum). Abbreviations: VA (vitamin A); WHR (waist–hip ratio); BMI (body mass index); BF% (body fat percentage); SF (skinfold); and EC (energy contribution). Statistical assessment of the differences between each genotype group were assessed by an ANOVA test adjusted for age.

## Supplementary Materials

The following are available online at https://www.mdpi.com/2072-6643/12/9/2588/s1. Table S1. Rs code, gene and chromosome (Chr) location of SNPs studied for their potential modulating effect on vitamin A metabolism.

## References

[1] Seidell J.C., Halberstadt J. (2015). The Global Burden of Obesity and the Challenges of Prevention. Ann. Nutr. Metab..

[2] Arroyo-Johnson C., Mincey K.D. (2016). Obesity Epidemiology Worldwide. Gastroenterol. Clin. N. Am..

[3] Wiseman E.M., Bar-El Dadon S., Reifen R. (2017). The vicious cycle of vitamin a deficiency: A review. Crit. Rev. Food Sci. Nutr..

[4] García O.P. (2012). Effect of vitamin A deficiency on the immune response in obesity. Proc. Nutr. Soc..

[5] Vaughan L.A., Benyshek D.C., Martin J.F. (1997). Food acquisition habits, nutrient intakes, and anthropometric data of Havasupai adults. J. Am. Diet. Assoc..

[6] Viroonudomphol D., Pongpaew P., Tungtrongchitr R., Changbumrung S., Tungtrongchitr A., Phonrat B., Vudhivai N., Schelp F.P. (2003). The relationships between anthropometric measurements, serum vitamin A and E concentrations and lipid profiles in overweight and obese subjects. Asia Pac. J. Clin. Nutr..

[7] García O., Ronquillo D., del Carmen Caamaño M., Martínez G., Camacho M., López V., Rosado J. (2013). Zinc, Iron and Vitamins A, C and E Are Associated with Obesity, Inflammation, Lipid Profile and Insulin Resistance in Mexican School-Aged Children. Nutrients.

[8] Bonet M.L., Ribot J., Galmés S., Serra F., Palou A. (2020). Carotenoids and carotenoid conversion products in adipose tissue biology and obesity: Pre-clinical and human studies. Biochim. Biophys. Acta Mol. Cell Biol. Lipids.

[9] Blomhoff R., Blomhoff H.K. (2006). Overview of retinoid metabolism and function. J. Neurobiol..

[10] Bonet M.L., Canas J.A., Ribot J., Palou A. (2015). Carotenoids and their conversion products in the control of adipocyte function, adiposity and obesity. Arch. Biochem. Biophys..

[11] Bonet M.L., Ribot J., Felipe F., Palou A. (2003). Vitamin A and the regulation of fat reserves. Cell. Mol. Life Sci..

[12] Bonet M.L., Ribot J., Palou A. (2012). Lipid metabolism in mammalian tissues and its control by retinoic acid. Biochim. Biophys. Acta Mol. Cell Biol. Lipids.

[13] Bonet M.L., Mercader J., Palou A. (2017). A nutritional perspective on UCP1-dependent thermogenesis. Biochimie.

[14] Amengual J., Petrov P., Bonet M.L., Ribot J., Palou A. (2012). Induction of carnitine palmitoyl transferase 1 and fatty acid oxidation by retinoic acid in HepG2 cells. Int. J. Biochem. Cell Biol..

[15] Eggersdorfer M., Wyss A. (2018). Carotenoids in human nutrition and health. Arch. Biochem. Biophys..

[16] Canas J.A., Lochrie A., McGowan A.G., Hossain J., Schettino C., Balagopal P.B. (2017). Effects of mixed carotenoids on adipokines and abdominal adiposity in children: A pilot study. J. Clin. Endocrinol. Metab..

[17] Beydoun M.A., Chen X., Jha K., Beydoun H.A., Zonderman A.B., Canas J.A. (2019). Carotenoids, vitamin A, and their association with the metabolic syndrome: A systematic review and meta-analysis. Nutr. Rev..

[18] Borel P., Desmarchelier C., Nowicki M., Bott R. (2015). A Combination of Single-Nucleotide Polymorphisms Is Associated with Interindividual Variability in Dietary -Carotene Bioavailability in Healthy Men. J. Nutr..

[19] Gueguen S., Leroy P., Gueguen R., Siest G., Visvikis S., Herbeth B. (2005). Genetic and environmental contributions to serum retinol and alpha-tocopherol concentrations: The Stanislas Family Study. Am. J. Clin. Nutr..

[20] Mondul A.M., Yu K., Wheeler W., Zhang H., Weinstein S.J., Major J.M., Cornelis M.C., Männistö S., Hazra A., Hsing A.W. (2011). Genome-wide association study of circulating retinol levels. Hum. Mol. Genet..

[21] Valacchi G., Sticozzi C., Lim Y., Pecorelli A. (2011). Scavenger receptor class B type I: A multifunctional receptor. Ann. N. Y. Acad. Sci..

[22] Mercader J., Ribot J., Murano I., Felipe F., Cinti S., Bonet M.L., Palou A. (2006). Remodeling of White Adipose Tissue after Retinoic Acid Administration in Mice. Endocrinology.

[23] Felipe F., Bonet M.L., Ribot J., Palou A. (2003). Up-regulation of muscle uncoupling protein 3 gene expression in mice following high fat diet, dietary vitamin A supplementation and acute retinoic acid-treatment. Int. J. Obes..

[24] Borel P., Moussa M., Reboul E., Lyan B., Defoort C., Vincent-Baudry S., Maillot M., Gastaldi M., Darmon M., Portugal H. (2007). Human Plasma Levels of Vitamin E and Carotenoids Are Associated with Genetic Polymorphisms in Genes Involved in Lipid Metabolism. J. Nutr..

[25] Ebrahimzadeh Attari V., Asghari Jafarabadi M., Zemestani M., Ostadrahimi A. (2015). Effect of Zingiber officinale Supplementation on Obesity Management with Respect to the Uncoupling Protein 1 -3826A > G and β3-adrenergic Receptor Trp64Arg Polymorphism. Phyther. Res..

[26] Snitker S., Fujishima Y., Shen H., Ott S., Pi-Sunyer X., Furuhata Y., Sato H., Takahashi M. (2009). Effects of novel capsinoid treatment on fatness and energy metabolism in humans: Possible pharmacogenetic implications. Am. J. Clin. Nutr..

[27] Bergman R.N., Stefanovski D., Buchanan T.A., Sumner A.E., Reynolds J.C., Sebring N.G., Xiang A.H., Watanabe R.M. (2011). A Better Index of Body Adiposity. Obesity.

[28] Ortega R.M., Andrés P., Requejo A.M., Aparicio A., Molinero L.M., López-Sobaler A.M. DIAL software for assessing diets and food calculations (for Windows, version 2.0). Department of Nutrition (UCM) & Alce Ingeniería, S.L. Madrid, Spain. https://www.alceingenieria.net/infodial.htm.

[29] European Food Safety Authority (EFSA) Dietary Reference Values: Vitamin A Advice Published|European Food Safety Authority. https://www.efsa.europa.eu/en/press/news/150305.

[30] Shen W.-J., Azhar S., Kraemer F.B. (2018). SR-B1: A Unique Multifunctional Receptor for Cholesterol Influx and Efflux. Annu. Rev. Physiol..

[31] Borel P., Lietz G., Goncalves A., Szabo de Edelenyi F., Lecompte S., Curtis P., Goumidi L., Caslake M.J., Miles E.A., Packard C. (2013). CD36 and SR-BI Are Involved in Cellular Uptake of Provitamin A Carotenoids by Caco-2 and HEK Cells, and Some of Their Genetic Variants Are Associated with Plasma Concentrations of These Micronutrients in Humans. J. Nutr..

[32] Murholm M., Isidor M.S., Basse A.L., Winther S., Sørensen C., Skovgaard-Petersen J., Nielsen M.M., Hansen A.S., Quistorff B., Hansen J.B. (2013). Retinoic acid has different effects on UCP1 expression in mouse and human adipocytes. BMC Cell Biol..

[33] Pierelli G., Stanzione R., Forte M., Migliarino S., Perelli M., Volpe M., Rubattu S. (2017). Uncoupling protein 2: A key player and a potential therapeutic target in vascular diseases. Oxid. Med. Cell. Longev..

[34] Serra F., Bonet M.L., Puigserver P., Oliver J., Palou A. (1999). Stimulation of uncoupling protein 1 expression in brown adipocytes by naturally occurring carotenoids. Int. J. Obes. Relat. Metab. Disord..

[35] Larose M., Cassard-Doulcier A.M., Fleury C., Serra F., Champigny O., Bouillaud F., Ricquier D. (1996). Essential cis-acting elements in rat uncoupling protein gene are in an enhancer containing a complex retinoic acid response domain. J. Biol. Chem..

[36] Bonet M.L., Puigserver P., Serra F., Ribot J., Vázquez F., Pico C., Palou A. (1997). Retinoic acid modulates retinoid X receptor alpha and retinoic acid receptor alpha levels of cultured brown adipocytes. FEBS Lett..

[37] Cadenas S. (2018). Mitochondrial uncoupling, ROS generation and cardioprotection. Biochim. Biophys. Acta Bioenerg..

[38] Constantineau J., Greason E., West M., Filbin M., Kieft J.S., Carletti M.Z., Christenson L.K., Rodriguez A. (2010). A synonymous variant in scavenger receptor, class B, type I gene is associated with lower SR-BI protein expression and function. Atherosclerosis.

[39] Nagai N., Sakane N., Tsuzaki K., Moritani T. (2011). UCP1 genetic polymorphism (–3826 A/G) diminishes resting energy expenditure and thermoregulatory sympathetic nervous system activity in young females. Int. J. Obes..

[40] Yoneshiro T., Ogawa T., Okamoto N., Matsushita M., Aita S., Kameya T., Kawai Y., Iwanaga T., Saito M. (2013). Impact of UCP1 and β3AR gene polymorphisms on age-related changes in brown adipose tissue and adiposity in humans. Int. J. Obes..

[41] Kovacs P., Ma L., Hanson R.L., Franks P., Stumvoll M., Bogardus C., Baier L.J. (2005). Genetic variation in UCP2 (uncoupling protein-2) is associated with energy metabolism in Pima Indians. Diabetologia.

[42] Galmés S., Cifre M., Palou A., Oliver P., Serra F. (2019). A Genetic Score of Predisposition to Low-Grade Inflammation Associated with Obesity May Contribute to Discern Population at Risk for Metabolic Syndrome. Nutrients.

[43] Reynés B., Díaz-Rúa R., Cifre M., Oliver P., Palou A. (2014). Peripheral blood mononuclear cells as a potential source of biomarkers to test the efficacy of weight-loss strategies. Obesity.

[44] Cifre M., Díaz-Rua R., Varela Calviño R., Reynés B., Pericás-Beltrán J., Palou A., Oliver P. (2016). Human peripheral blood mononuclear cell in vitro system to test the efficacy of food bioactive compounds: Effects of polyunsaturated fatty acids and their relation with BMI. Mol. Nutr. Food Res..

[45] European Food Safety Authority (EFSA) (2017). Dietary Reference Values for nutrients Summary report. EFSA Support. Publ..

[46] Ruiz E., Ávila J.M., Valero T., del Pozo S., Rodriguez P., Aranceta-Bartrina J., Gil Á., González-Gross M., Ortega R.M., Serra-Majem L. (2015). Energy Intake, Profile, and Dietary Sources in the Spanish Population: Findings of the ANIBES Study. Nutrients.

[47] European Food Safety Authority (EFSA) (2013). Scientific Opinion on Dietary Reference Values for energy. EFSA J..

[48] Harari A., Coster A.C., Jenkins A., Xu A., Greenfield J.R., Harats D., Shaish A., Samocha-Bonet D. (2020). Obesity and Insulin Resistance Are Inversely Associated with Serum and Adipose Tissue Carotenoid Concentrations in Adults. J. Nutr..

[49] Borel P., Desmarchelier C. (2017). Genetic Variations Associated with Vitamin A Status and Vitamin A Bioavailability. Nutrients.

[50] Morabia A., Ross B.M., Costanza M.C., Cayanis E., Flaherty M.S., Alvin G.B., Das K., James R., Yang A.-S., Evagrafov O. (2004). Population-based study of SR-BI genetic variation and lipid profile. Atherosclerosis.

[51] Ma R., Zhu X., Yan B. (2018). SCARB1 rs5888 gene polymorphisms in coronary heart disease: A systematic review and a meta-analysis. Gene.

[52] Ye L.-F., Zheng Y.-R., Zhang Q.-G., Yu J.-W., Wang L.-H. (2017). Meta-analysis of the association between SCARB1 polymorphism and fasting blood lipid levels. Oncotarget.

[53] Qian L., Xu K., Xu X., Gu R., Liu X., Shan S., Yang T. (2013). UCP2 -866G/A, Ala55Val and UCP3 -55C/T polymorphisms in association with obesity susceptibility—A meta-analysis study. PLoS ONE.

[54] Andersen G., Dalgaard L.T., Justesen J.M., Anthonsen S., Nielsen T., Thørner L.W., Witte D., Jørgensen T., Clausen J.O., Lauritzen T. (2013). The frequent UCP2 −866G > A polymorphism protects against insulin resistance and is associated with obesity: A study of obesity and related metabolic traits among 17 636 Danes. Int. J. Obes..

[55] Lee M., Chae S., Cha Y., Park Y. (2012). Supplementation of Korean fermented soy paste doenjang reduces visceral fat in overweight subjects with mutant uncoupling protein-1 allele. Nutr. Res..

[56] Dixon J., O’Brien P. (2006). Obesity and the White Blood Cell Count: Changes with Sustained Weight Loss. Obes. Surg..

[57] Koca T.T. (2017). Does obesity cause chronic inflammation? The association between complete blood parameters with body mass index and fasting glucose. Pak. J. Med. Sci..

[58] Reynés B., Priego T., Cifre M., Oliver P., Palou A. (2018). Peripheral Blood Cells, a Transcriptomic Tool in Nutrigenomic and Obesity Studies: Current State of the Art. Compr. Rev. Food Sci. Food Saf..

[59] Picó C., Serra F., Rodríguez A.M., Keijer J., Palou A. (2019). Biomarkers of Nutrition and Health: New Tools for New Approaches. Nutrients.

[60] Laurencikiene J., Rydé M. (2012). Liver X receptors and fat cell metabolism. Int. J. Obes..

[61] Anderson C.M., Stahl A. (2013). SLC27 fatty acid transport proteins. Mol. Asp. Med..

[62] Caimari A., Oliver P., Rodenburg W., Keijer J., Palou A. (2010). Slc27a2 expression in peripheral blood mononuclear cells as a molecular marker for overweight development. Int. J. Obes..

[63] Becuwe P., Ennen M., Klotz R., Barbieux C., Grandemange S. (2014). Manganese superoxide dismutase in breast cancer: From molecular mechanisms of gene regulation to biological and clinical significance. Free Radic. Biol. Med..

[64] Grdina D.J., Murley J.S., Miller R.C., Mauceri H.J., Sutton H.G., Thirman M.J., Li J.J., Woloschak G.E., Weichselbaum R.R. (2013). A Manganese Superoxide Dismutase (SOD2)-Mediated Adaptive Response. Radiat. Res..

[65] Ulven S.M., Dalen K.T., Gustafsson J.-Å., Nebb H.I. (2005). LXR is crucial in lipid metabolism. Prostaglandins Leukot. Essent. Fat. Acids.

[66] Liu K., Yeh I., Chou S., Yen M., Kuo P. (2018). Regulatory mechanism of fatty acid-CoA metabolic enzymes under endoplasmic reticulum stress in lung cancer. Oncol. Rep..

[67] Oishi Y., Spann N.J., Link V.M., Muse E.D., Strid T., Edillor C., Kolar M.J., Matsuzaka T., Hayakawa S., Tao J. (2017). SREBP1 Contributes to Resolution of Pro-inflammatory TLR4 Signaling by Reprogramming Fatty Acid Metabolism. Cell Metab..

[68] Rahman S.M., Janssen R.C., Choudhury M., Baquero K.C., Aikens R.M., De La Houssaye B.A., Friedman J.E. (2012). CCAAT/enhancer-binding protein β (C/EBPβ) expression regulates dietary-induced inflammation in macrophages and adipose tissue in mice. J. Biol. Chem..

[69] Baker R.G., Hayden M.S., Ghosh S. (2011). NF-κB, inflammation, and metabolic disease. Cell Metab..

[70] Zhang R., Wang Y., Li R., Chen G. (2015). Transcriptional Factors Mediating Retinoic Acid Signals in the Control of Energy Metabolism. Int. J. Mol. Sci..

[71] Goodarzi M.O. (2018). Genetics of obesity: What genetic association studies have taught us about the biology of obesity and its complications. Lancet Diabetes Endocrinol..

