# Supplementary material for: Increased Risk of High Body Fat and Altered Lipid Metabolism Associated to Suboptimal Consumption of Vitamin A Is Modulated by Genetic Variants rs5888 (SCARB1), rs1800629 (UCP1) and rs659366 (UCP2)
Source: Nutrients. 2020 Aug 26;12(9):2588. doi: 10.3390/nu12092588 (PMC7551832; doi:10.3390/nu12092588)
Supplement: Supplementary file 1 [file nutrients-12-02588-s001.pdf]

Table S1. Rs code, gene and chromosome (Chr) location of SNPs studied for their potential modulating effect on vitamin A metabolism.

Rs Code *Gene* (Chromosome)	Gene-vitamin A Metabolism Relationship	Meaning of the Nucleotideexchange	Metabolic Risk Associated with Genotype
**rs5888** ***SCARB1*** (Chr: 12)	*SCARB1* gene product (SR-B1) is a multifunctional scavenger involved in dietary/blood carotenoid uptake/transport [1]	1. Synonym amino acid exchange (A350A) on exon 8;<br>2. Potential changes in the secondary structure of its mRNA (allele T) [2];<br>3. Lower *in vitro* SR-B1 protein expression (allele T cell carriers), but not in transcript levels [2].	Allele T is associated with:<br>1. Lower hydrophobic antioxidant (α-tocopherol and β-cryptoxanthin) plasma levels [3];<br>2. In men, lower risk of coronary heart disease in a meta-analysis [4]. Probably, because of lower triglycerides [5] and higher HDL-c plasma levels [6];<br>3. ncreased risk of premature coronary artery disease, specifically, in women [1].
**rs1800592** ***UCP1*** (Chr: 4)	Carotenoids and retinoids enhance fatty acid oxidation and promotes UCP1-associated thermogenesis in rodents [7,8]	1. Located on *UCP1* promoter (-A3826G) region;<br>2. Greater *UCP1* expression and thermogenic capacity (allele A) [9,10].	Allele A is associated with:<br>1. Greater effect of specific bio-active compounds of Zingiber on body fat reduction, in AA genotype carriers [11].<br>1. Less frequent in obese subjects [12].
**rs659366** ***UCP2*** (Chr: 11)	Vitamin A supplementation increases *UCP2* expression in adipose tissue in rodents [8,13]	1. Located on *UCP2* promoter (-C866T) region;<br>2. Greater *UCP2* expression (allele T) [14].	Allele T is associated with:<br>1. Greater effect of specific bio-active compound (capsaicin) on abdominal fat reduction [15].<br>2. Lower risk of obesity [16].<br>3. Lower insulin resistance risk (HOMA index) [17];

References

1. Valacchi, G.; Sticozzi, C.; Lim, Y.; Pecorelli, A. Scavenger receptor class B type I: a multifunctional receptor. *Ann. N. Y. Acad. Sci.* **2011**, *1229*, E1–E7, doi:10.1111/j.1749-6632.2011.06205.x.
2. Constantineau, J.; Greason, E.; West, M.; Filbin, M.; Kieft, J. S.; Carletti, M. Z.; Christenson, L. K.; Rodriguez, A. A synonymous variant in scavenger receptor, class B, type I gene is associated with lower SR-BI protein expression and function. *Atherosclerosis* **2010**, *210*, 177–182, doi:10.1016/j.atherosclerosis.2009.11.029.
3. Borel, P.; Moussa, M.; Reboul, E.; Lyan, B.; Defoort, C.; Vincent-Baudry, S.; Maillot, M.; Gastaldi, M.; Darmon, M.; Portugal, H.; Planells, R.; Lairon, D. Human Plasma Levels of Vitamin E and Carotenoids Are Associated with Genetic Polymorphisms in Genes Involved in Lipid Metabolism. *J. Nutr.* **2007**, *137*, 2653–2659, doi:10.1093/jn/137.12.2653.
4. Ma, R.; Zhu, X.; Yan, B. SCARB1 rs5888 gene polymorphisms in coronary heart disease: A systematic review and a meta-analysis. *Gene* **2018**, *678*, 280–287, doi:10.1016/j.gene.2018.08.024.

5. Ye, L.-F.; Zheng, Y.-R.; Zhang, Q.-G.; Yu, J.-W.; Wang, L.-H. Meta-analysis of the association between SCARB1 polymorphism and fasting blood lipid levels. *Oncotarget* **2017**, *8*, 81145–81153, doi:10.18632/oncotarget.20867.
6. Xie, L.; Lv, X.; Sun, Y.; Tong, Y.; Zhang, S.; Deng, Y. Association of rs5888 SNP in SCARB1 gene with coronary artery disease. *Herz* **2019**, *44*, 644–650, doi:10.1007/s00059-018-4689-z.
7. Mercader, J.; Ribot, J.; Murano, I.; Felipe, F.; Cinti, S.; Bonet, M. L.; Palou, A. Remodeling of White Adipose Tissue after Retinoic Acid Administration in Mice. *Endocrinology* **2006**, *147*, 5325–5332, doi:10.1210/en.2006-0760.
8. Bonet, M. L.; Ribot, J.; Galmés, S.; Serra, F.; Palou, A. Carotenoids and carotenoid conversion products in adipose tissue biology and obesity: Pre-clinical and human studies. *Biochim. Biophys. Acta - Mol. Cell Biol. Lipids* **2020**, 158676, doi:10.1016/j.bbalip.2020.158676.
9. Nagai, N.; Sakane, N.; Tsuzaki, K.; Moritani, T. UCP1 genetic polymorphism (–3826 A/G) diminishes resting energy expenditure and thermoregulatory sympathetic nervous system activity in young females. *Int. J. Obes.* **2011**, *35*, 1050–1055, doi:10.1038/ijo.2010.261.
10. Yoneshiro, T.; Ogawa, T.; Okamoto, N.; Matsushita, M.; Aita, S.; Kameya, T.; Kawai, Y.; Iwanaga, T.; Saito, M. Impact of UCP1 and β3AR gene polymorphisms on age-related changes in brown adipose tissue and adiposity in humans. *Int. J. Obes.* **2013**, *37*, 993–998, doi:10.1038/ijo.2012.161.
11. Ebrahimzadeh Attari, V.; Asghari Jafarabadi, M.; Zemestani, M.; Ostadrahimi, A. Effect of Zingiber officinale Supplementation on Obesity Management with Respect to the Uncoupling Protein 1 -3826A>G and β3-adrenergic Receptor Trp64Arg Polymorphism. *Phyther. Res.* **2015**, *29*, 1032–1039, doi:10.1002/ptr.5343.
12. Chathoth, S.; Ismail, M. H.; Vatte, C.; Cyrus, C.; Al Ali, Z.; Ahmed, K. A.; Acharya, S.; Al Barqi, A. M.; Al Ali, A. Association of Uncoupling Protein 1 (UCP1) gene polymorphism with obesity: a case-control study. *BMC Med. Genet.* **2018**, *19*, 203, doi:10.1186/s12881-018-0715-5.
13. Felipe, F.; Bonet, M. L.; Ribot, J.; Palou, A. Up-regulation of muscle uncoupling protein 3 gene expression in mice following high fat diet, dietary vitamin A supplementation and acute retinoic acid-treatment. *Int. J. Obes.* **2003**, *27*, 60–69, doi:10.1038/sj.ijo.0802188.
14. Kovacs, P.; Ma, L.; Hanson, R. L.; Franks, P.; Stumvoll, M.; Bogardus, C.; Baier, L. J. Genetic variation in UCP2 (uncoupling protein-2) is associated with energy metabolism in Pima Indians. *Diabetologia* **2005**, *48*, 2292–5, doi:10.1007/s00125-005-1934-9.
15. Snitker, S.; Fujishima, Y.; Shen, H.; Ott, S.; Pi-Sunyer, X.; Furuhata, Y.; Sato, H.; Takahashi, M. Effects of novel capsinoid treatment on fatness and energy metabolism in humans: possible pharmacogenetic implications. *Am. J. Clin. Nutr.* **2009**, *89*, 45–50, doi:10.3945/ajcn.2008.26561.
16. Qian, L.; Xu, K.; Xu, X.; Gu, R.; Liu, X.; Shan, S.; Yang, T. UCP2 -866G/A, Ala55Val and UCP3 -55C/T polymorphisms in association with obesity susceptibility - a meta-analysis study. *PLoS One* **2013**, *8*, e58939, doi:10.1371/journal.pone.0058939.
17. Andersen, G.; Dalgaard, L. T.; Justesen, J. M.; Anthonsen, S.; Nielsen, T.; Thørner, L. W.; Witte, D.; Jørgensen, T.; Clausen, J. O.; Lauritzen, T.; Holmkvist, J.; Hansen, T.; Pedersen, O. The frequent UCP2 −866G>A polymorphism protects against insulin resistance and is associated with obesity: a study of obesity and related metabolic traits among 17 636 Danes. *Int. J. Obes.* **2013**, *37*, 175–181, doi:10.1038/ijo.2012.22.
